# Fetal growth restriction followed by very preterm birth is associated with smaller kidneys but preserved kidney function in adolescence

**DOI:** 10.1007/s00467-022-05785-x

**Published:** 2022-11-21

**Authors:** Jonas Liefke, Caroline Heijl, Katarina Steding-Ehrenborg, Eva Morsing, Håkan Arheden, David Ley, Erik Hedström

**Affiliations:** 1 Clinical Physiology, Department of Clinical Sciences Lund, Lund University, Skåne University Hospital, Lund, Sweden; 2 Cardiology, Department of Clinical Sciences Lund, Lund University, Skåne University Hospital, Lund, Sweden; 3 Paediatrics, Department of Clinical Sciences Lund, Lund University, Skåne University Hospital, Lund, Sweden; 4 Diagnostic Radiology, Department of Clinical Sciences Lund, Lund University, Skåne University Hospital, Lund, Sweden

**Keywords:** Very preterm, Fetal growth restriction, Adolescence, Kidney volume, Kidney parenchyma, Renal cortical volume, Renal medullary volume, Angiotensinogen, Kidney function

## Abstract

**Background:**

Preterm birth and fetal growth restriction (FGR) are associated with structural and functional kidney changes, increasing long-term risk for chronic kidney disease and hypertension. However, recent studies in preterm children are conflicting, indicating structural changes but normal kidney function. This study therefore assessed kidney structure and function in a cohort of adolescents born very preterm with and without verified FGR.

**Methods:**

Adolescents born very preterm with FGR and two groups with appropriate birthweight (AGA) were included; one matched for gestational week at birth and one born at term. Cortical and medullary kidney volumes and T1 and T2* mapping values were assessed by magnetic resonance imaging. Biochemical markers of kidney function and renin–angiotensin–aldosterone system (RAAS) activation were analyzed.

**Results:**

Sixty-four adolescents were included (13–16 years; 48% girls). Very preterm birth with FGR showed smaller total (66 vs. 75 ml/m^2^; *p* = 0.01) and medullary volume (19 vs. 24 ml/m^2^; *p* < 0.0001) compared to term AGA. Corticomedullary volume ratio decreased from preterm FGR (2.4) to preterm AGA (2.2) to term AGA (1.9; p = 0.004). There were no differences in T1 or T2* values (all *p* ≥ 0.34) or in biochemical markers (all *p* ≥ 0.12) between groups.

**Conclusions:**

FGR with abnormal fetal blood flow followed by very preterm birth is associated with smaller total kidney and medullary kidney volumes, but not with markers of kidney dysfunction or RAAS activation in adolescence. Decreased total kidney and medullary volumes may still precede a long-term decrease in kidney function, and potentially be used as a prognostic marker.

**Graphical abstract:**

**Figure:**
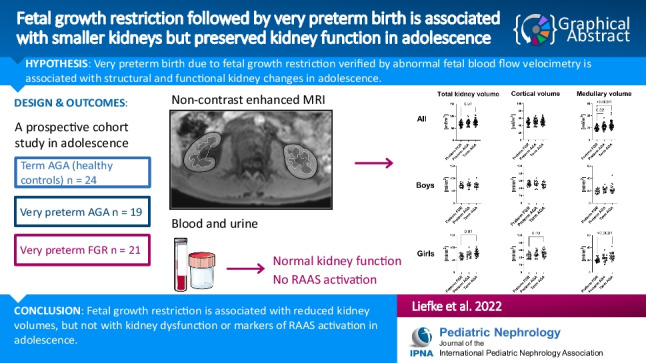
A higher resolution version of the Graphical abstract is available as Supplementary information

**Supplementary Information:**

The online version contains supplementary material available at 10.1007/s00467-022-05785-x.

## Introduction

Preterm birth and fetal growth restriction (FGR) are both known to affect kidney development with a reduction in number of nephrons and of total kidney volume [1–4]. This increases risk for chronic kidney disease and hypertension [5, 6], which can be linked to biochemical markers of kidney function and renin–angiotensin–aldosterone (RAAS) activation [7]. However, recent studies performed during childhood following very preterm birth show conflicting results, indicating structural kidney changes with a preserved kidney function and normal blood pressure [8–10]. Previous studies with the objective of relating FGR to health outcomes have mainly used birthweight as a proxy for FGR. This has led to inclusion of constitutionally small infants as well as truly growth-restricted infants [11, 12]. This can be avoided by verifying the pathophysiology of early-onset FGR leading to very preterm birth by measuring fetal blood flow abnormalities using Doppler ultrasound [13]. Further, sex has been suggested as a modifying factor to that of preterm birth with impact on both kidney structure and function, with smaller kidney volumes and decreased kidney function in females [14–16].

Total kidney volume is commonly assessed with ultrasound [8, 9], and although separate quantification of cortical and medullary parenchyma in the preterm child has been reported [17], ultrasound consistently underestimates kidney volumes and has high intra- and interobserver variability [18]. Magnetic resonance imaging (MRI) more accurately quantifies kidney volumes [18] and enables separate renal cortical and medullary volume quantification without contrast agents [19]. Further, non-invasive tissue assessment of renal interstitial fibrosis and hypoxia can be performed with T1 and T2* mapping by MRI [20]. Renal interstitial fibrosis and hypoxia are common pathways toward chronic kidney disease independent of cause [21, 22], and renal parenchymal T1 and T2* might therefore be affected after preterm birth and FGR.

We hypothesized that very preterm birth due to FGR verified by abnormal fetal blood flow velocimetry is associated with structural and functional kidney changes in adolescence. The specific aims were to study the impact of very preterm birth and FGR on (1) renal cortical and medullary volumes and renal T1 and T2* values as determined by MRI, (2) biochemical markers of kidney function and RAAS activation, and (3) possible modifying effects by sex.

## Methods

### Study population

This study was conducted at Skåne University Hospital, Lund, Sweden, between 2014 and 2019. The Regional Ethical Review board in Lund, Sweden, approved the study (Dnr 2013/244) and all participants and their guardians when appropriate provided written informed consent before participation. Participants underwent MRI, and blood and urine were sampled for biochemical analyses. Body surface area (BSA) was calculated using the Mosteller formula [23].

The current study is part of a prospective cohort study of subjects actively delivered very preterm (< 30 gestational weeks) after early-onset FGR, with birth weight > 2 standard deviations below the mean according to the Swedish national standard [24] and with absent or reversed end-diastolic flow in the umbilical artery as determined by a standardized protocol using Doppler velocimetry according to guidelines [13]. Between 1998 and 2004, 42 such live-born infants were delivered on fetal indication at Skåne University Hospital in Lund, Sweden. Thirty-four of these infants were available for follow-up examinations in childhood (*preterm FGR)*. Two control groups with birth weight appropriate for gestational age (AGA) were identified. The first control group consisted of a subgroup (*n* = 42) of all infants (*n* = 371) admitted to the neonatal intensive care unit in Lund during the same time period as the preterm FGR group [25]. This subgroup was matched to the index group for sex, gestational age at birth and year of birth (*preterm AGA*). The second control group, born vaginally after a healthy pregnancy, was included in childhood and matched to the index group for sex and year of birth (*term AGA*). Characteristics at birth, and perinatal and maternal morbidity, as well as follow-up studies and neuro-cognitive, cardiovascular and pulmonary outcomes, have been reported previously [25–28]. All adolescents in the three groups that were studied in childhood were now asked to participate in the current study in adolescence.

### Magnetic resonance imaging

Participants underwent non-contrast-enhanced MRI using either a 1.5 T Philips Achieva (Best, the Netherlands), or a 1.5 T Magnetom Aera (Siemens Healthineers, Erlangen, Germany). For quantification of kidney volumes, in vivo fast low-angle shot (FLASH) MR images were acquired as transaxial stacks during breath-hold, covering the entire kidneys [19]. Typical parameters were 1.3 × 1.3 × 6 mm, TR/TE = 152/5.57 ms, FA = 80°, bandwidth = 270 Hz/px, GRAPPA = 2 with 24 reference lines, water excitation and 50-mm saturation bands head/foot with gap 10 mm.

Renal coronal T1 maps were based on a modified Look-Locker inversion recovery (MOLLI) sequence using a 5(3)3 scheme, with typical parameters: 1.4 × 1.4 × 8 mm, TR/TE = 281/1.12 ms, FA = 35°. Renal coronal T2* maps were based on a multi-echo gradient recalled echo sequence using 10 echoes with first echo 1.07 ms and step 1.36 ms (i.e., range 3–13.3 ms). Typical parameters were 3.1 × 3.1 × 8 mm, TR = 200 ms, FA = 20°.

A 2D phase-contrast gradient recalled echo sequence with retrospective ECG gating was used for quantitative flow measurements. Typical image parameters for the flow measurements in the ascending aorta and descending aorta at diaphragm level were TR/TE = 9.84/2.67 ms; flip angle = 20°; in-plane resolution 1.5 × 1.5 × 5 mm and VENC 200 cm/s. Typical image parameters for flow measurements in the renal arteries were TR/TE = 9.84/2.67 ms; flip angle = 20°; in-plane resolution 1.5 × 1.5 × 5 mm and VENC 100 cm/s.

### MR image analysis

Images were analyzed in Segment (version 3.0, http://segment.heiberg.se) [29].

One blinded observer with 6 years (JL) of MRI experience performed measurements of kidney volumes, blood flow, and T1 and T2* mapping. Data were confirmed in consensus with a blinded pediatric radiologist with 22 years of MRI experience (EH). Intra- and interobserver variability for renal parenchymal volume quantification has previously been reported [19].

### Kidney volumes

**Fig. 1:**
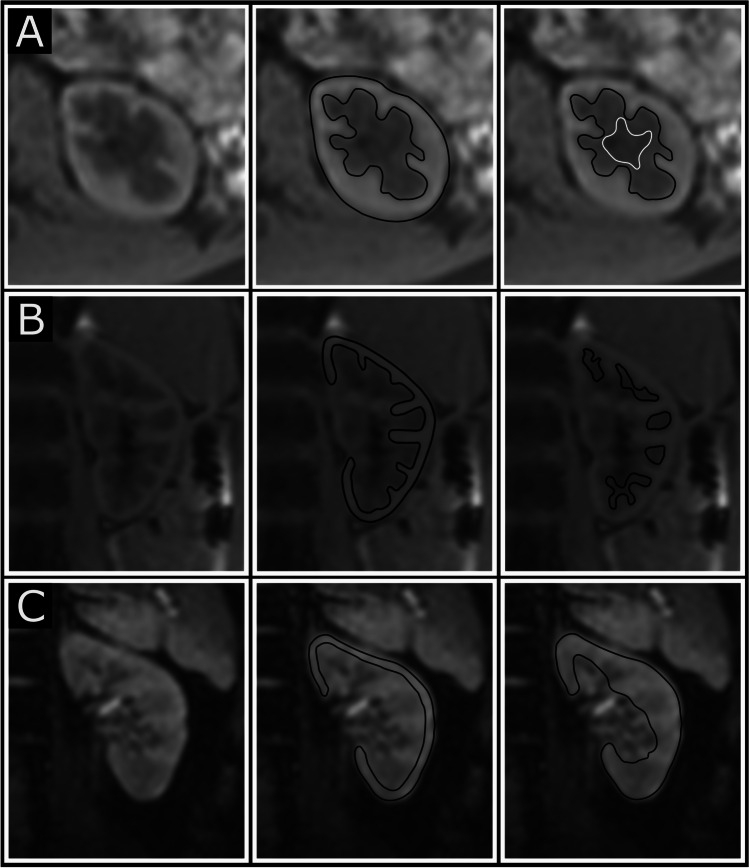
MR images with delineations for renal cortical and medullary volumes (**A**), and for renal parenchymal T1 (**B**) and T2* (**C**) values. A transaxial image of the left kidney for volume measurements without delineations (**A**; left), and with cortical (**A**; middle) and medullary delineations (**A**; right). A coronal image of the left kidney for T1 mapping measurements without delineations (**B**; left), and with cortical (**B**; middle) and medullary delineations (**B;** right). A coronal image of the left kidney for T2* measurements without delineations (**C**; left), and with cortical (**C**; middle) and total parenchymal delineations (**C**; right). Renal parenchymal borders are depicted by black lines, and non-parenchymal tissue removed from volume (**A**) are depicted by white lines. For T1 and T2* values delineations were placed to avoid partial volume effects. Both left and right kidneys were delineated

Cortex and medulla were manually delineated for volumes, and cysts, renal pelvis, and other non-parenchymal tissue excluded (Fig. 1) as previously described [19]. In short, borders of cortex and medulla were delineated in transversal slices covering the entire kidney. Cortex and medulla volumes were then calculated by adding the respective slice volumes, as previously validated [19]. Corticomedullary volume ratio was calculated as cortical volume divided by medullary volume. Total kidney volume was defined as the combination of cortical and medullary volumes, excluding renal pelvis, blood vessels, and other non-parenchymal structures. Kidney volumes were normalized to BSA as growth pattern in those born preterm with and without low birth weight differs from birth up to young adulthood [30].

### Flow measurements

Quantitative flow measurements were performed in the ascending aorta, descending aorta at diaphragm level and in renal arteries. Flow measurements were performed in order to calculate filtration fraction, a measure of renal flow reserve [31], and because T2* is dependent on the inflow of oxygenated blood [20]. Vessels were delineated in magnitude images throughout the cardiac cycle using an automated algorithm with manual correction as needed and guided by phase-contrast images where appropriate [32]. Linear background phase correction was performed [33]. Kidney parenchymal perfusion was calculated as renal arterial blood flow divided by total kidney mass (density 1.05 g per 1 ml). As hematocrit was not available, glomerular filtration fraction was calculated as estimated glomerular filtration rate (eGFR) divided by renal blood flow multiplied with 0.55 in boys and with 0.6 in girls to give an estimation of the fraction of plasma in whole blood for boys and girls, respectively [34].

### Renal T1 and T2* mapping

T1 maps were generated using motion-correction and a 3-parameter T1 fit with Look-Locker correction while T2* maps were generated using a 2-parameter mono-exponential fit [35]. To ensure tissue homogeneity, the region of interest (ROI) was drawn one pixel in from the outer contour of the respective parenchymal area and for cortical ROIs the renal column was not delineated all the way down to the renal pelvis (Fig. 1). T1 values were acquired for renal cortex and medulla separately. Corticomedullary T1 differentiation, a marker of kidney dysfunction [36], was calculated as the ratio between cortical and medullary T1. T2* values were acquired for the renal cortex separately and for the entire renal parenchyma combined (cortex and medulla) as it was not possible to accurately distinguish between cortical columns and medulla in all echo images that were used for creating T2* maps (Fig. 1). Although acquired during breath-hold, minor motion made ROI transfer between echo images uncertain for T2* quantification.

### Biochemical markers

Due to potential structural and functional kidney alterations following very preterm birth and FGR, both standard clinical and new biochemical markers of kidney function and RAAS activation were assessed.

Blood and urine were acquired in conjunction with MRI and were not guaranteed to be first-morning samples. Urine and blood samples were collected in sterile test tubes and EDTA test tubes, respectively, and directly centrifuged (Thermo Scientific Megafuge 8, Thermo Fisher Scientific, Waltham, USA) at 1500 G for 10 min, pipetted into cryotubes and stored at –80 °C.

Plasma concentrations of cystatin C, creatinine, and renin as well as urine samples of creatinine, albumin, IgG, kappa, and lambda were quantified at the hospital laboratory. Cystatin C concentration was quantified using a Atellica Solution immunoassay analyzer (Siemens Healthineers, Erlangen, Germany) with a coefficient of variation of 0.7% (quantification limit 0.25–8.93 mg/L). Plasma renin concentration was quantified using an automated Chemiluminescence Immunoassay (CLIA) (WHO International Standard for Direct Renin, 68/356), with coefficient of variation 8–10% (< 98 mIE/L) [37]. In short, samples were incubated together with two monoclonal antibodies coated with acridinium and biotin, respectively. Streptavidin magnetic particles were added and after washing and adding of trigger reagents, the light emitted by the acridinium-labeled antibody was used to determine renin concentration.

Urine was analyzed using standard protein electrophoresis, with quantification of creatinine, albumin, IgG, kappa, and lambda. Coefficients of variation were for creatinine 4%, for albumin 8% at 15 mg/L and 4% at 50 mg/L and for the other analytes 5%.

Urine angiotensinogen (u-AGT), a potential biochemical marker of intra-renal RAAS activation [38], was quantified using a solid-phase sandwich enzyme-linked immunosorbent assay (ELISA) (Human Total Angiotensinogen Assay Kit-IBL, Cat. no. 27412, 1091–1 Naka Aza-Higashida, Fujioka-Shi, Gunma 375–0005, Japan) on an ELISA absorbance microplate reader (Infinite F50 with software Magellan 7.2, Tecan Trading AG, Switzerland) with a sensitivity of 0.03 ng/mL and a specificity of 100%. Four samples had been remeasured and thus thawed twice, and as angiotensinogen is sensitive to temperature changes these four samples were removed from further analysis.

The ratios of u-IgG, u-albumin, u-kappa, u-lambda, and u-AGT to u-creatinine were calculated. Estimated glomerular filtration rate was calculated as per clinical routine for children and adolescents (< 18 years of age), using the Caucasian, Asian, Pediatric and Adult (CAPA) equation based on Cystatin C (eGFR_Cystatin C_) [39]. For comparison, the updated Schwartz equation based on creatinine (eGFR_Creatinine_) [40] and the average of eGFR_Cystatin C_ and eGFR_Creatinine_ (eGFR_Average_) were assessed.

### Statistical analyses

Statistical analyses were performed using SPSS 26.0 (IBM Corp, Armonk, NY, USA) and GraphPad Prism 9 (GraphPad Software, La Jolla, California, USA). Data are expressed as median (range) unless otherwise specified. Kruskal–Wallis with post hoc Bonferroni’s or Dunn’s multiple comparison test assessed group differences and Mann–Whitney *U*-test assessed sex differences. The Jonckheere–Terpsta and Kendall’s Tau-b tests were used for assessing trends in kidney volumes between groups. Pearson’s chi square test or Fisher’s exact test was used for categorical variables as appropriate. All statistical tests were two-sided and *p* values < 0.05 were considered to show statistically significant differences.

## Results

### Study population

Seventy-one adolescents from the original cohort agreed to participate in the current study. Seven participants were excluded due to incomplete image acquisition related to aborted scans on the participants’ request. Thus, 64 adolescents were included in the current study. Table 1 shows characteristics at birth and adolescence for the respective groups. Of the 102 participants in the original cohort, one individual was too old (19 years) to be included, three individuals were unreachable, and 27 chose not to participate at follow-up. Of those who underwent MRI, nine individuals did not consent to sampling of blood whereas six individuals did not consent to sampling of urine (Table 4).

**Table 1:** Characteristics at birth and adolescence

	Preterm FGR *n* = *21*	Preterm AGA *n* = *19*	Term AGA *n* = *24*	*P*			
				Between all groups	Preterm FGR vs. preterm AGA	Preterm FGR vs. term AGA	Preterm AGA vs. term AGA
Characteristics at birth							
Gestational age at birth (weeks + days)	26 + 6 (24 + 4–29 + 1)	27 + 4 (24 + 3 – 29 + 5)	40 (38 + 3 – 40 + 5)	< 0.0001	1	< 0.0001	< 0.0001
Birthweight (g)	660 (395–976)	1110 (660 – 1790)	3445 (3000 – 4160)	< 0.0001	0.02	< 0.0001	< 0.0001
Birthweight deviation (%)	–34.1(–62.7 to –22.5)	–1.7 (–13.6 to 14.3)	–2.15 (–16.8 to 21.5)	< 0.0001	< 0.0001	< 0.0001	1
Characteristics in adolescence							
Age (years)	15 (13 – 16)	15 (13 – 16)	15 (13 – 16)	0.88			
Girls (n (%))	11 (52)	9 (47)	13 (54)	0.9			
Weight (kg)	52 (38 – 90)	57 (37 – 74)	58 (37 – 89)	0.54			
Length (cm)	160 (150 – 180)^*^	168 (149 – 183)	167 (155 – 189)	0.051			
BMI (kg/m^2^)	19 (15 – 28)	20 (15 – 24)	20 (15 – 25)	0.89			
BSA (m^2^)	1.5 (1.3 – 2.1)	1.6 (1.2 – 1.9)	1.7 (1.3 – 2.2)	0.31			
Systolic blood pressure (mmHg)	105 (87 – 123)	107 (88 – 120)	102 (89 – 130)	0.43			
Diastolic blood pressure (mmHg)	53 (41 – 69)	54 (45 – 65)	50 (44 – 80)	0.22			

Data are presented as median (range). A single asterisk indicates a difference between preterm FGR (158 (150–165) cm) and term AGA (163 (157–176) cm; *p* = 0.02) for girls. *FGR* fetal growth restriction, *AGA* appropriate for gestational age

### Magnetic resonance imaging

#### Kidney volumes

**Fig. 2:**
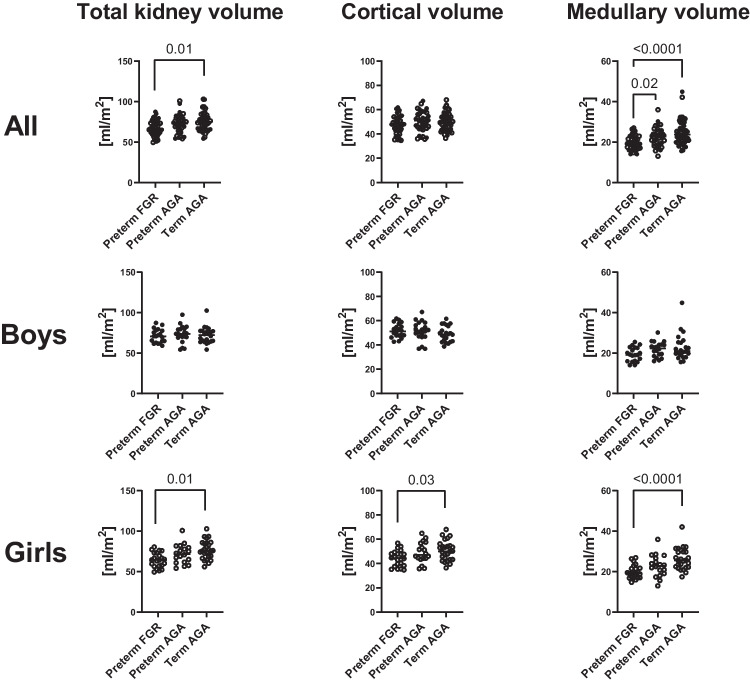
Kidney volumes normalized for body surface area. Total kidney volumes (left column), cortical volumes (middle column), and medullary volumes (right column) are shown for both sexes combined (upper row), boys (middle row), and girls (lower row) in adolescents born preterm with fetal growth restriction (preterm FGR), preterm with birth weight appropriate for gestational age (preterm AGA), and born at term (term AGA). Girls born preterm due to FGR had smaller BSA-adjusted parenchymal volumes compared to girls born term AGA. FGR, fetal growth restriction; AGA, appropriate for gestational age

Table 2 and Fig. 2 show renal parenchymal volumes in absolute and in BSA-adjusted values. The preterm FGR group had a smaller median medullary volume (19 vs. 23 ml/m^2^; *p* = 0.02) as compared to the preterm AGA group, and a smaller median total kidney volume (66 vs. 75 ml/m^2^; *p* = 0.01) and medullary volume (19 vs. 24 ml/m^2^; *p* < 0.0001) as compared to the term AGA group. Trend analyses showed a decrease in median total kidney volume (75 vs. 73 vs. 66 ml/m^2^; *p* = 0.02) and medullary volume (24 vs. 23 vs. 19 ml/m^2^; *p* = 0.001) from term AGA to preterm AGA to preterm FGR. The corticomedullary volume ratio was higher in the preterm FGR group as compared to the term AGA group (2.4 vs. 1.9; *p* = 0.01), whereas no group differences were observed in cortical volumes (*p* = 0.34). Trend analyses showed a decrease in corticomedullary volume ratio from preterm FGR (2.4) to preterm AGA (2.2) to term AGA (1.9; *p* = 0.004) for both sexes combined. A significant trend was also observed in boys (*p* = 0.02) and in girls (*p* = 0.04), respectively (Table 2 and Fig. 2).

When stratifying for sex, girls born preterm FGR had smaller median total kidney volume (65 vs. 76 ml/m^2^; *p* = 0.001), smaller cortical volume (44 vs. 50 ml/m^2^; *p* = 0.03), and smaller medullary volume (19 vs. 26 ml/m^2^; *p* < 0.0001) as compared to the term AGA group. Further, in girls, trend analyses showed for preterm FGR vs. preterm AGA vs. term AGA smaller total (65 vs. 72 vs. 76 ml/m^2^; *p* = 0.004), cortical (44 vs. 47 vs. 50 ml/m^2^; *p* = 0.045), and medullary (19 vs. 23 vs. 26 ml/m^2^; *p* = 0.001) volumes. For boys, there were no corresponding group differences in kidney volumes after adjustment for BSA (all *p* ≥ 0.08).

In the preterm FGR group, girls had smaller median total kidney volume (95 vs. 116 ml; *p* = 0.049), cortical volume (66 vs. 86 ml; *p* = 0.003), and BSA-adjusted cortical volume (44 vs. 51 ml/m^2^; *p* = 0.02) compared to boys. There were no sex differences in either absolute or BSA-adjusted kidney volumes in the preterm AGA or term AGA group (all *p* ≥ 0.13).

#### Flow measurements

Table 3 shows blood flow measurements in the ascending aorta, descending aorta at diaphragm level and renal arteries. There were no group differences in aortic or renal blood flow (all *p* ≥ 0.19). Glomerular filtration fraction was similar between groups (all *p* ≥ 0.12).

**Table 2:** Kidney volumes and parenchymal T1 and T2* values

				*P*				
	Preterm	Preterm	Term	Between groups	Preterm FGR vs. preterm AGA	Preterm FGR vs. term AGA	Preterm AGA vs. term AGA	Trend across groups
	FGR	AGA	AGA					
All	*n* = *21*	*n* = *19*	*n* = *24*					
Total parenchymal volume (ml)	105 (66–161)	121 (74–180)	122 (80–177)	**0.004**	0.06	**0.004**	> 0.99	**0.02**
Total parenchymal volume (ml/m^2^)	66 (50–87)	73 (54–101)	75 (54–103)	**0.01**	0.12	**0.01**	> 0.99	**0.02**
Cortical volume (ml)	77 (45–114)	84 (49–124)	82 (52–133)	0.1	N/A	N/A	N/A	0.24
Cortical volume (ml/m^2^)	48 (35–62)	51 (36–67)	50 (36–68)	0.34	N/A	N/A	N/A	0.41
Medullary volume (ml)	30 (20–48)	36 (20–57)	38 (27–71)	**< 0.0001**	**0.01**	**< 0.0001**	0.2	**0.0002**
Medullary volume (ml/m^2^)	19 (14–27)	23 (13–34)	24 (16–45)	**< 0.0001**	**0.02**	**< 0.0001**	0.33	**0.001**
Corticomedullary ratio	2.41 (1.97–3.51)	2.23 (1.62–3.14)	1.91 (1.47 – 2.95)	**0.018**	0.77	**0.014**	0.34	**0.004**
Boys	*n* = *10*	*n* = *10*	*n* = *11*					
Total parenchymal volume (ml)	116 (80–161)	125 (74–180)	124 (94–177)	0.31	N/A	N/A	N/A	0.45
Total parenchymal volume (ml/m^2^)	71 (59–87)	74 (54–97)	72 (54–103)	0.7	N/A	N/A	N/A	0.66
Cortical volume (ml)	86 (60–114)	87 (49–124)	85 (66–133)	0.54	N/A	N/A	N/A	0.91
Cortical volume (ml/m^2^)	51 (43–62)	52 (37–67)	49 (39–62)	0.34	N/A	N/A	N/A	0.33
Medullary volume (ml)	30 (20–47)	38 (25–56)	37 (27–71)	**0.02**	0.1	**0.03**	> 0.99	0.0503
Medullary volume (ml/m^2^)	19 (14–25)	22 (16–30)	21 (16–45)	0.08	N/A	N/A	N/A	0.14
Corticomedullary ratio	2.79 (2.1–3.5)	2.28 (1.95–3.1)	2.2 (1.47–2.95)	0.07	N/A	N/A	N/A	**0.02**
Girls	*n* = *11*	*n* = *9*	*n* = *13*					
Total parenchymal volume (ml)	95 (66–140)	106 (82–160)	121 (80–154)	**0.002**	**0.021**	**0.001**	0.37	**0.008**
Total parenchymal volume (ml/m^2^)	65 (50–80)	72 (54–101)	76 (56–103)	**0.002**	0.15	**0.001**	0.53	**0.004**
Cortical volume (ml)	66 (45–92)	70 (51–103)	80 (52–104)	**0.02**	0.22	**0.02**	> 0.99	**0.041**
Cortical volume (ml/m^2^)	44 (35–57)	47 (36–65)	50 (36–68)	**0.03**	0.18	**0.03**	> 0.99	**0.045**
Medullary volume (ml)	29 (21–48)	33 (20–57)	39 (27–63)	**< 0.0001**	0.23	**< 0.0001**	0.054	**0.001**
Medullary volume (ml/m^2^)	19 (15–27)	23 (13–36)	26 (17–42)	**0.0001**	0.17	**< 0.0001**	0.13	**0.001**
Corticomedullary ratio	2.09 (1.97–2.64)	2.07 (1.62–3.13)	1.81 (1.51–2.64)	0.1	N/A	N/A	N/A	**0.038**
T1 values	*n* = *19*	*n* = *19*	*n* = *22*					
T1 cortex (ms)	1032 (927–1311)	1056 (926–1137)*	1034 (970–1165)	0.34	N/A	N/A	N/A	0.50
T1 medulla (ms)	1574 (1478–1712)	1568 (1455–1703)	1600 (1389–1692)	0.39	N/A	N/A	N/A	0.56
Corticomedullary T1 differentiation (%)	64 (54–71)	67 (62–73)*	65 (58–81)	0.08	N/A	N/A	N/A	0.62
T2* values	*n* = *21*	*n* = *19*	*n* = *21*					
T2* cortex (ms)	76 (70–90)	79 (64–83)	76 (63–84)	0.51	N/A	N/A	N/A	0.37
T2* parenchyma (ms)	76 (68–88)	77 (63–87)	77 (65–87)	0.99	N/A	N/A	N/A	0.89

Data presented as median (range). Renal parenchymal volumes are presented in absolute and BSA-adjusted values. A single asterisk indicates differences between groups for girls (data presented in Results). *FGR* fetal growth restriction, *AGA* appropriate for gestational age

#### Renal T1 and T2* mapping

Table 2 shows renal T1 and T2* mapping values. There were no differences in renal T1 or T2* mapping values between groups (all *p* ≥ 0.34) or for corticomedullary T1 differentiation (all p ≥ 0.08). When stratifying for sex, median cortical T1 was higher in girls in the preterm AGA group compared to the preterm FGR group (1086 vs. 1037 ms; *p* = 0.02) and to the term AGA group (1086 vs. 1027 ms; *p* = 0.03). Further, in girls, median corticomedullary T1 differentiation was higher in the preterm AGA group compared to the preterm FGR group (67 vs. 64%; *p* = 0.005). In boys, no group differences were observed (all *p* ≥ 0.29).

**Table 3:** Blood flow measurements

	Preterm FGR	Preterm AGA	Term AGA	*P* between groups
	*n* = *21*	*n* = *19*	*n* = *24*	
Cardiac output ascending aorta (L/min)	5.3 (2.8–7.4)	5.8 (3.1–9.2)	5.8 (4.0–9.5)	0.5
Cardiac index ascending aorta (L/min/m^2^)	3.5 (2.0–4.9)	3.7 (2.2–5.0)	3.6 (2.6–5.2)	0.74
Cardiac output abdominal aorta (L/min)	3.3 (1.9–4.6)	3.6 (1.7–5.9)	3.7 (2.6–6.4)	0.48
Cardiac index abdominal aorta (L/min/m^2^)	2.1 (1.3–2.7)	2.1 (1.2–3.2)	2.2 (1.4–3.1)	0.47
Renal arterial blood flow (L/min)	0.8 (0.5–1.2)	0.9 (0.6–1.1)	0.9 (0.7–1.2) ^n = 22^	0.19
Renal blood flow/cardiac output (%)	15 (10–20)	14 (10–20)	15 (10–20) ^n = 22^	0.95
Renal blood flow/BSA (L/min/m^2^)	0.5 (0.4–0.7)	0.5 (0.4–0.7)	0.5 (0.4–0.7) ^n = 22^	0.56
Filtration fraction (eGFR _Cystatin C_) (%)	19 (11–31) ^n = 17^	20 (16–36) ^n = 15^	19 (14–27) ^n =21^	0.75
Filtration fraction (eGFR _Creatinine_) (%)	21 (13–31) ^n = 17^	18 (13–27) ^n = 15^	18 (14–34) ^n = 21^	0.12
Filtration fraction (eGFR _Average_) (%)	20 (12–38) ^n = 17^	18 (17–28) ^n = 15^	18 (15–31) ^n = 21^	0.25
Total parenchymal blood flow (ml/min/g)	3.8 (2.7–6.2)	3.9 (2.8–4.6)	3.7 (2.6–5.0) ^n = 22^	0.81

Data are presented as median (range). Filtration fraction was calculated as the respective eGFR divided by total renal blood flow multiplied by 0.55 for boys and with 0.6 for girls. *FGR* fetal growth restriction, *AGA* appropriate for gestational age

#### Biochemical markers

Table 4 shows biochemical markers of kidney function and RAAS activation. No differences were observed between groups (all *p* ≥ 0.051). When stratifying for sex, boys in the preterm AGA group showed a lower median IgG/creatinine index compared to the term AGA group (0.35 vs. 0.78 g/mol; *p* = 0.04).

**Table 4:** Biochemical markers

	Preterm FGR	Preterm AGA	Term AGA	*P* between groups	Reference values
	*n* = *17*	*n = 15*	*n = 23*		
eGFR _(Cystatin C)_ ml/min/1.73 m^2^	91 (68–114)	102 (76–133)	100 (76–122)	0.2	86–134
eGFR _(Creatinine)_ ml/min/1.73 m^2^	97 (76–127)	89 (64–126)	99 (74–140)	0.31	86–134
eGFR _(Average)_ ml/min/1.73 m^2^	92 (81–119)	92 (75–127)	99 (85–125)	0.46	86–134
P-Renin [mIE/L]	31 (10–74)	28 (6–68)	31 (8–72)	0.75	5–80 (morning sample)
	*n* = *19*	*n* = *17*	*n* = *23*		
U-albumin/creatinine [g/mol]	0.61 (0.13–7.35)	0.34 (0.08–7.53)	0.9 (0.05–14.54)	0.09	< 3.0 (morning sample)
U-albumin/creatinine [mg/g]	54 (11–650)	30 (7–666)	79 (5–1285)	0.09	< 30 (morning sample)
U-IgG/creatinine [g/mol]	0.43 (0.16–2.11)	0.33 (0.18–0.76)*	0.57 (0.23–2.56)	0.051	< 0.8
U-Kappa/creatinine [g/mol]	0.09 (0.03–0.57)	0.12 (0.03–0.59)	0.14 (0.07–0.84)	0.14	< 0.6
U-Lambda/creatinine [g/mol]	0.01 (0–0.18)	0.02 (0–0.06)	0.02 (0–0.3)	0.64	< 0.6
U-AGT/creatinine ([ng/ml/ [µmol/L])	0.73 (0.24–2.29)	0.48 (0–1.6)	0.55 (0–7.5)	0.53	N/A

Data are presented as median (range). A single asterisk indicates lower values for boys born preterm AGA compared to term AGA (0.35 (0.23–0.74) vs. 0.78 (0.23–1.74); *p* = 0.04. *GFR* glomerular filtration rate, *IgG* immunoglobulin gamma, *AGT* angiotensinogen, *FGR* fetal growth restriction, *AGA* appropriate for gestational age

## Discussion

The current study shows that FGR followed by very preterm birth is associated with smaller total kidney and medullary kidney volumes in adolescence. Biomarkers of kidney function, kidney structure, and biochemical markers of RAAS activation were normal in adolescence after very preterm birth irrespective of preceding FGR.

### Kidney volumes

The current study shows smaller total kidney volumes and medullary volumes in adolescent age in subjects who experienced FGR resulting in very preterm birth. The differences were more pronounced in girls, showing smaller absolute and BSA-adjusted cortical and medullary volumes compared to term AGA. The current study is the first to assess the respective effects of very preterm birth in itself, and very preterm birth due to FGR on cortical and medullary volumes, respectively. It thus clarifies the impact of FGR and preterm birth respectively on kidney volumes in adolescence, and also confirms previously suggested sex differences, with smaller kidneys in girls [8, 14].

Although very preterm birth and FGR both associate with a low nephron count at birth [2, 4] and smaller kidney volumes across ages [7, 14], it is not possible to conclude that the smaller kidney volumes observed in the current study are a direct marker of nephron count. It is further not known whether smaller kidney volume per se represents pathology and thereby would be causal in increasing future risk for kidney disease, although such associations have been observed, especially in girls [7, 14, 15].

Kidney volumes decreased from term AGA to preterm AGA to preterm FGR, indicating that very preterm birth in itself, but more so in combination with FGR, affects kidney volumes in adolescence. Differences in kidney volumes between the preterm groups may be due to physiological changes related to placental insufficiency in FGR such as redistribution of fetal blood flow, hypoxia and nutritive impairment, previously associated with permanent changes and increased vulnerability in several organs, including the kidneys [1, 2, 41]. Further, several interventions associated with neonatal intensive care may have a negative impact on kidney function, at least in the short term [42], but with less knowledge about long-term effects. As indicated by the current results, it may be hypothesized that placental insufficiency, leading to early-onset FGR, sensitizes the kidneys to the adverse effects of neonatal intensive care, also leading to a decrease in kidney volumes in adolescence.

The renal cortex has been shown to undergo an accelerated postnatal hypertrophy in the preterm infant [17]. The increased corticomedullary volume ratio observed in the preterm groups in the current study, although mostly driven by smaller medullary volumes, could thus potentially indicate cortical hypertrophy in the preterm FGR group. A low nephron count at birth, as shown in the preterm infant [4], has been hypothesized to result in glomerular and cortical hypertrophy and to increase the risk for glomerular sclerosis and subsequent hypertrophy of the remaining glomeruli [43, 44]. The current study did not clearly show sclerosis by T1 mapping measurements after very preterm birth or FGR. However, it is well known that native T1 values in general are insensitive to structural tissue changes and that extracellular volume needs to be determined for accurate definition of sclerosis [45]. In the current study extracellular volume could however not be determined as contrast agent was not administered.

### Biomarkers of kidney structure and function and RAAS activation

Renal parenchymal T1 and T2* values as well as biochemical markers of kidney function and RAAS activation were similar between groups in the current study. Earlier studies have suggested that higher cortical T1 and lower corticomedullary T1 differentiation associate with chronic kidney disease and hypertension [36, 46], whereas RAAS activation after preterm birth has been linked to increased blood pressure and kidney dysfunction [7]. As both biochemical markers of kidney function and blood pressure were normal in the current study (Table 1) it is not surprising that renal parenchymal T1 and T2* values and biochemical markers of RAAS activation were within normal levels. However, when stratifying for sex, the current study showed higher cortical T1 and higher corticomedullary T1 differentiation in girls born preterm AGA compared to girls born preterm FGR. There were however no signs of kidney dysfunction or differences in renal blood flow or parenchymal changes affecting T2* that directly or indirectly could explain these findings.

Whether the observed changes in kidney volumes, corticomedullary volume ratio, and corticomedullary T1 differentiation in the current study are signs of girls being more susceptible for future functional decline and a “second hit” remains to be studied. An increased vulnerability in adolescent girls could however be hypothesized as female sex has been shown to increase the albumin/creatinine ratio after preterm birth [16] and to associate with smaller kidney volumes and decreased kidney function in middle-aged women born preterm [14, 15].

Although kidney volumes are commonly assessed with ultrasound, that method is prone to underestimation and has high variability [47], limiting accuracy and posing a risk to the patient if volume assessment guides treatment. The added value of accurate and separate kidney parenchymal assessment provided by MRI, together with short image acquisition time (< 5 min) make up for potential higher costs. To guide treatment, MRI for kidney volume assessment could be considered, especially in serial assessment of disease progression for pediatric populations and for populations with decreased kidney function, in all of which radiation and nephrotoxic contrast agents should be avoided.

### Limitations

Not all individuals from the original cohort accepted to participate in the current study, reducing statistical power. However, assessment of kidney volume using MRI has higher accuracy and precision than ultrasound [18], limiting a potential power issue. The study population was chosen dependent on a specific exposure and although strict inclusion criteria and matching was performed, direct causality cannot be inferred and the study is thus descriptive in its nature. Hydration status and sodium intake was not controlled and may impact T1 and T2* values. However, this has likely been similar between groups. Hematocrit was not available for calculating the filtration fraction. To limit potential sex-dependent errors in filtration fraction, separate estimates of plasma fraction were used for boys and girls. Further, most studies validating eGFR calculations based on cystatin C or creatinine use pediatric cohorts with known or probable kidney impairment. However, a wide range of glomerular filtration rates have been investigated and especially the cystatin C-based method shows low bias versus iohexol plasma clearance [39, 48]. Due to logistics, non-fasting samples of blood and urine were acquired. Descriptive statistics for the biochemical markers are reported for each group but variability within groups may be due to circadian rhythms rather than true differences or pathology.

### Conclusions

Fetal growth restriction with abnormal fetal blood flow followed by very preterm birth is associated with smaller total kidney and medullary kidney volumes, but not with markers of kidney dysfunction or RAAS activation in adolescence. Decreased total kidney and medullary volumes may still precede a long-term decrease in kidney function, and potentially be used as a prognostic marker.

## Supplementary Information

Below is the link to the electronic supplementary material.

Graphical Abstract (425 KB)

## Data Availability

The datasets generated and/or analyzed during the current study are not publicly available due to sensitive information but are available in anonymized form from the corresponding author on reasonable request.
