# Supplementary material for: Fetal growth restriction followed by very preterm birth is associated with smaller kidneys but preserved kidney function in adolescence
Source: Pediatr Nephrol. 2022 Nov 21;38(6):1855–66. doi: 10.1007/s00467-022-05785-x (PMC10154253; doi:10.1007/s00467-022-05785-x)
Supplement: Supplementary file 1 — Graphical Abstract (425 KB) [file 467_2022_5785_MOESM1_ESM.pptx]

## Slide 1
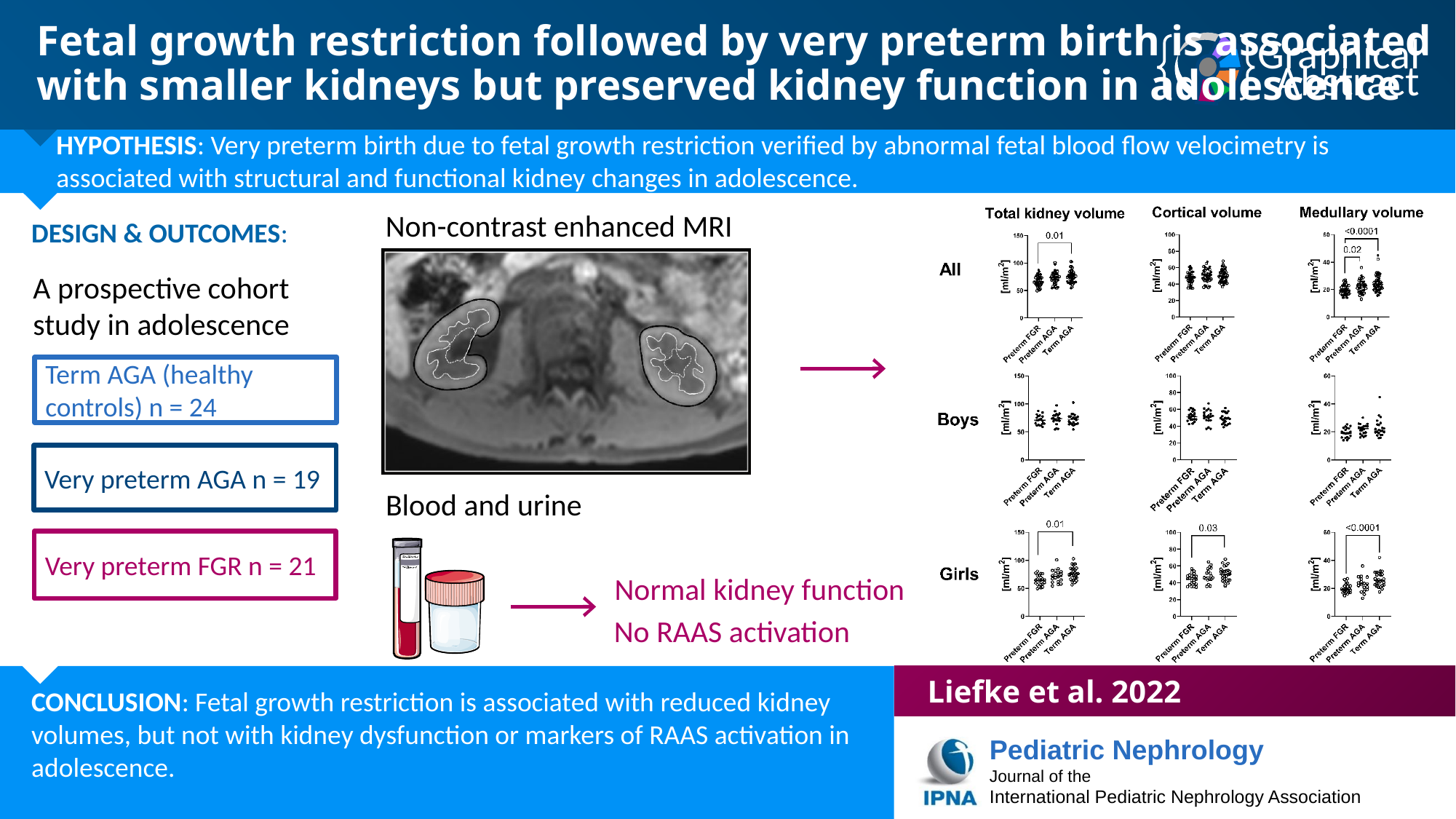

Fetal growth restriction followed by very preterm birth is associated
with smaller kidneys but preserved kidney function in adolescence
HYPOTHESIS: Very preterm birth due to fetal growth restriction verified by abnormal fetal blood flow velocimetry is
associated with structural and functional kidney changes in adolescence.
Non-contrast enhanced MRI
DESIGN & OUTCOMES:
A prospective cohort
study in adolescence
Term AGA (healthy controls) n = 24
Very preterm AGA n = 19
Blood and urine
Very preterm FGR n = 21
Normal kidney function
No RAAS activation
Liefke et al. 2022
CONCLUSION: Fetal growth restriction is associated with reduced kidney volumes, but not with kidney dysfunction or markers of RAAS activation in adolescence.
